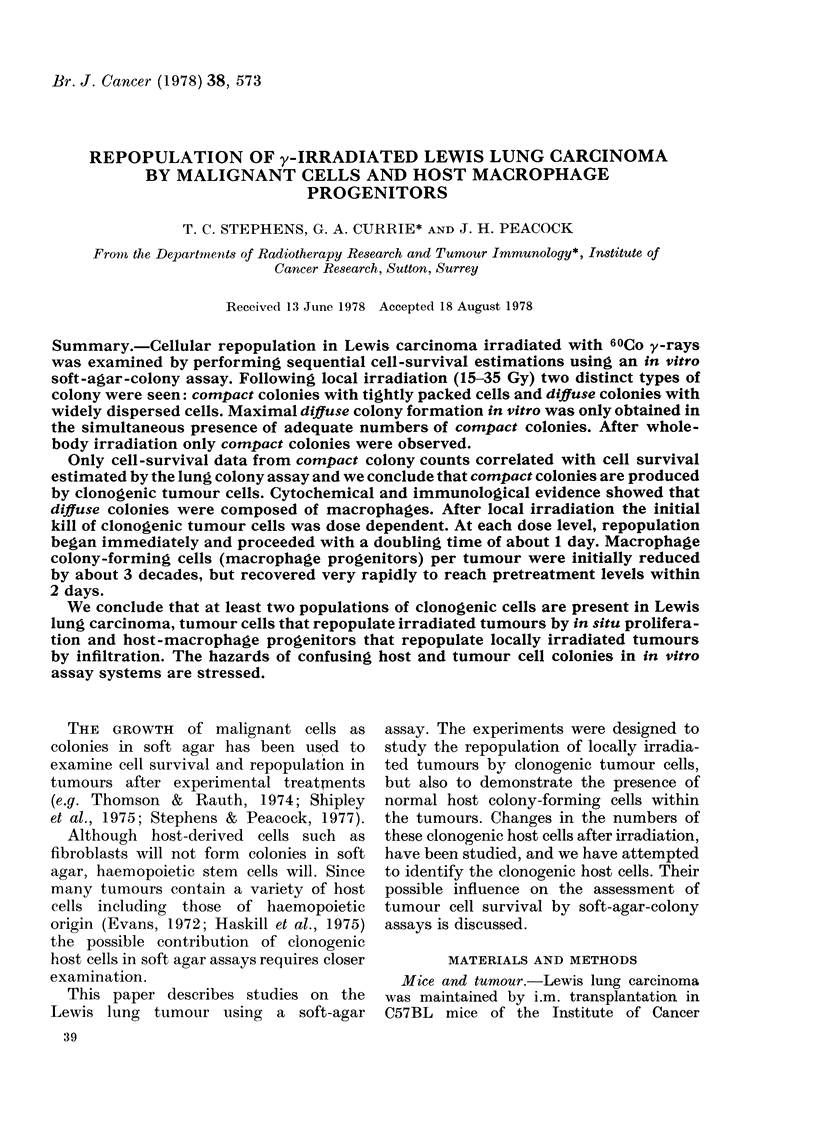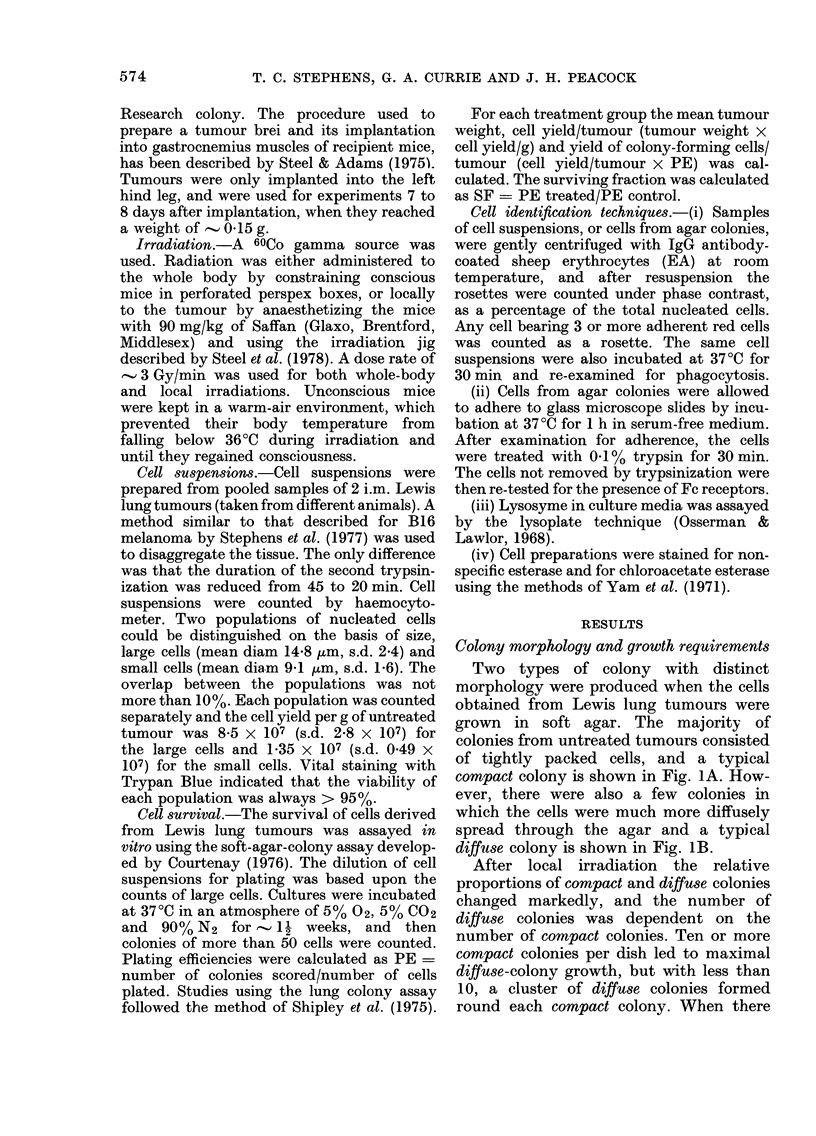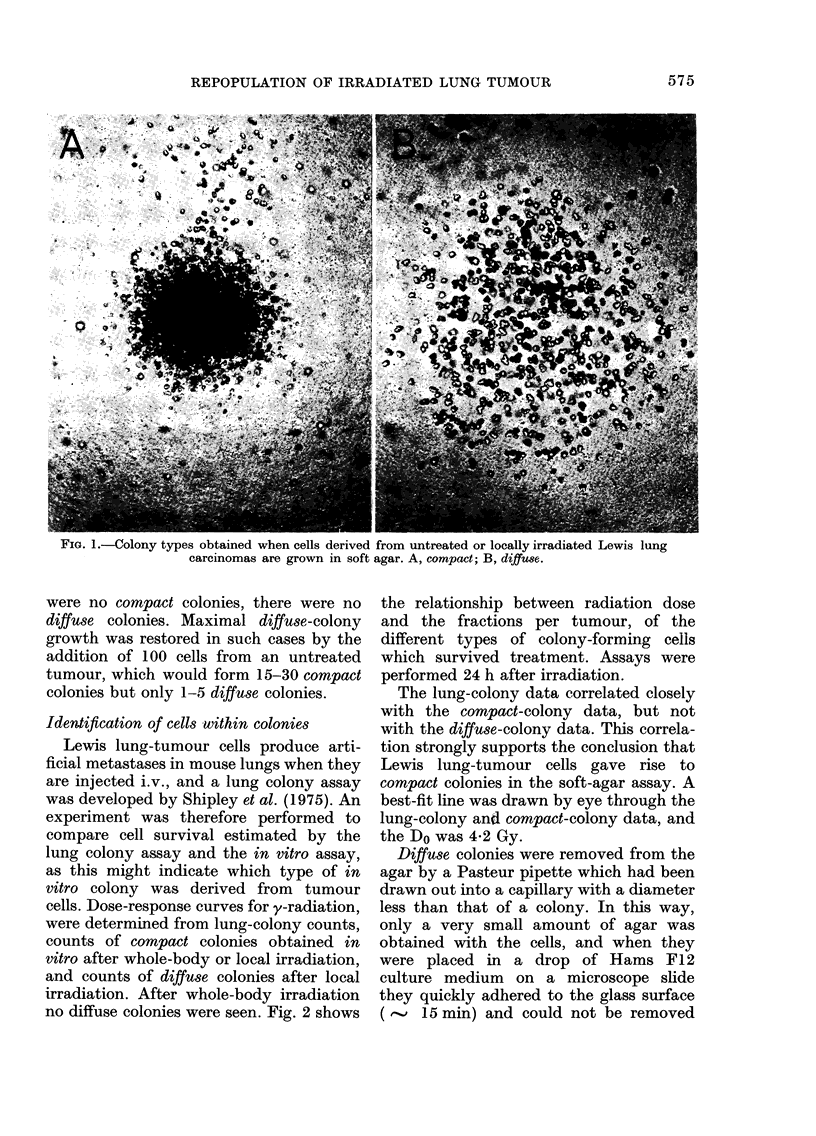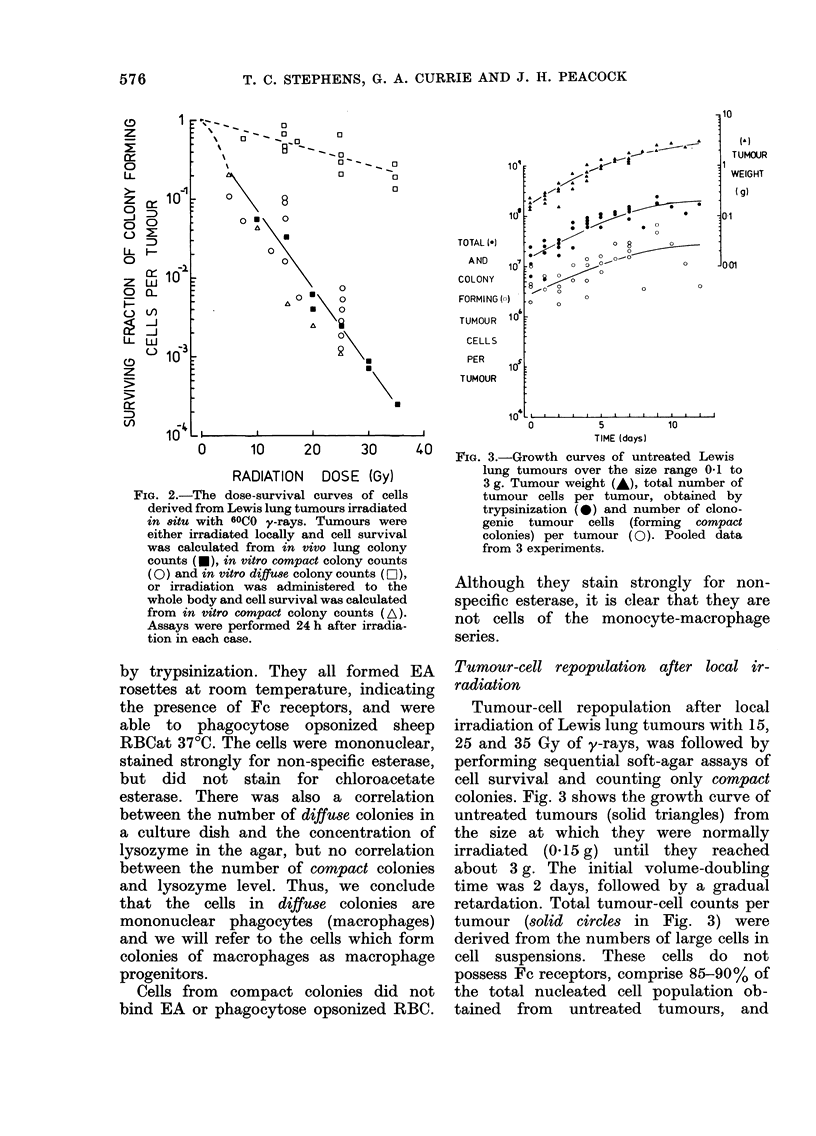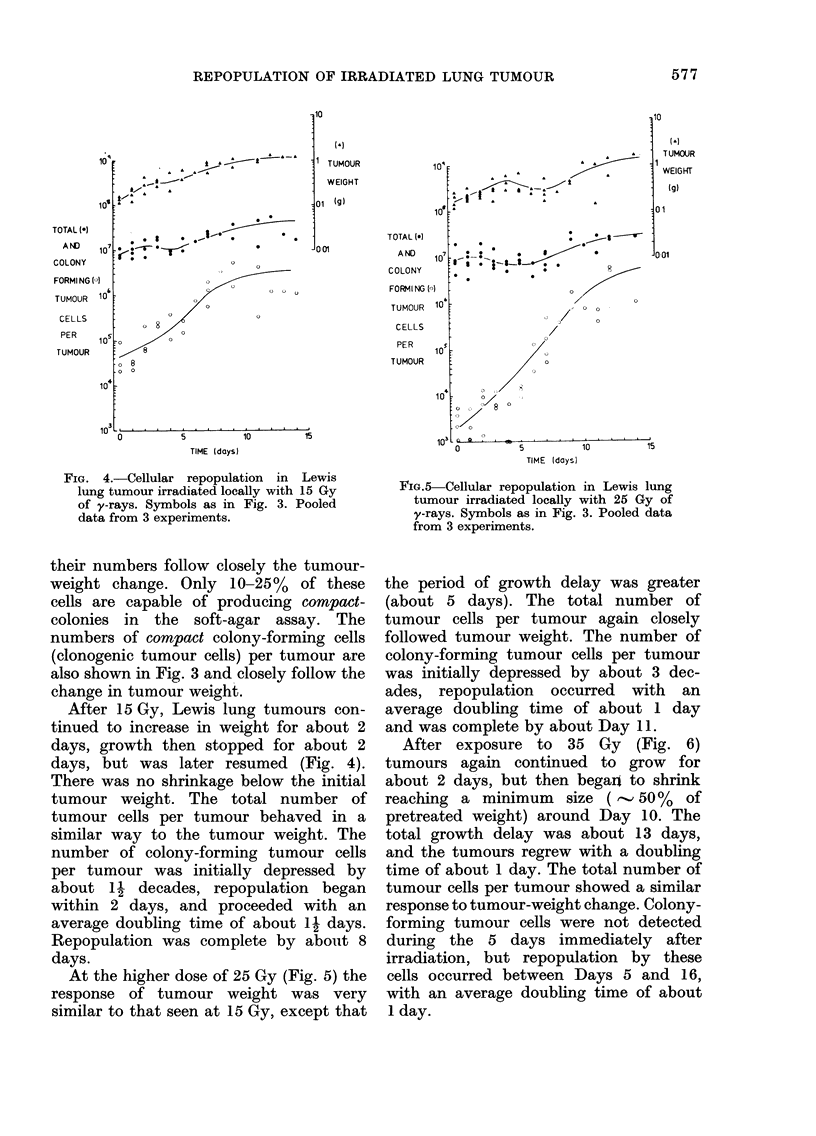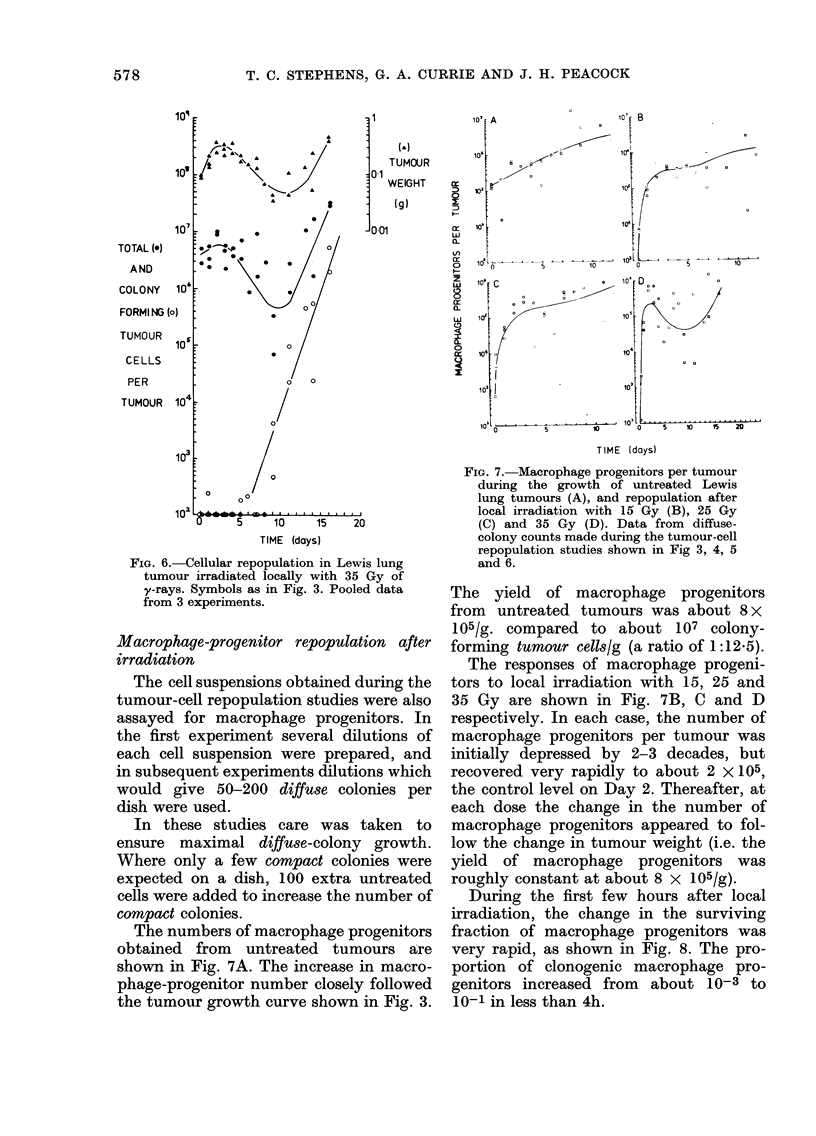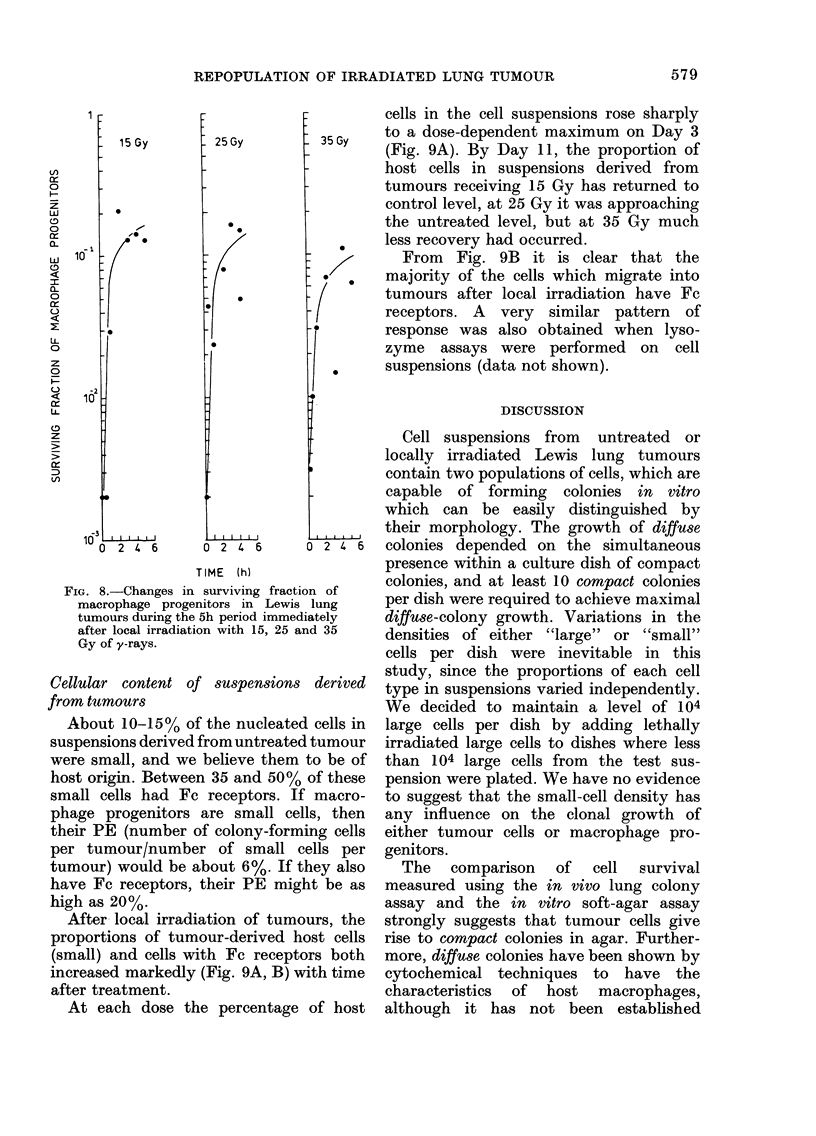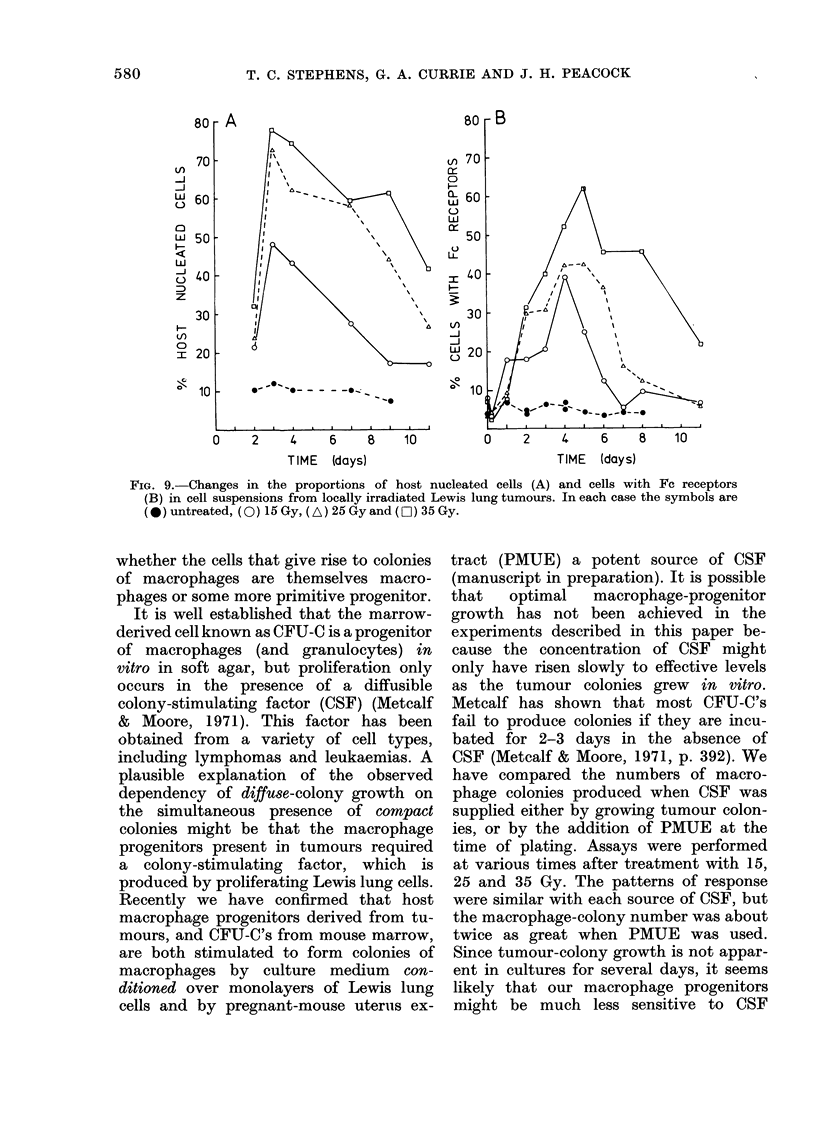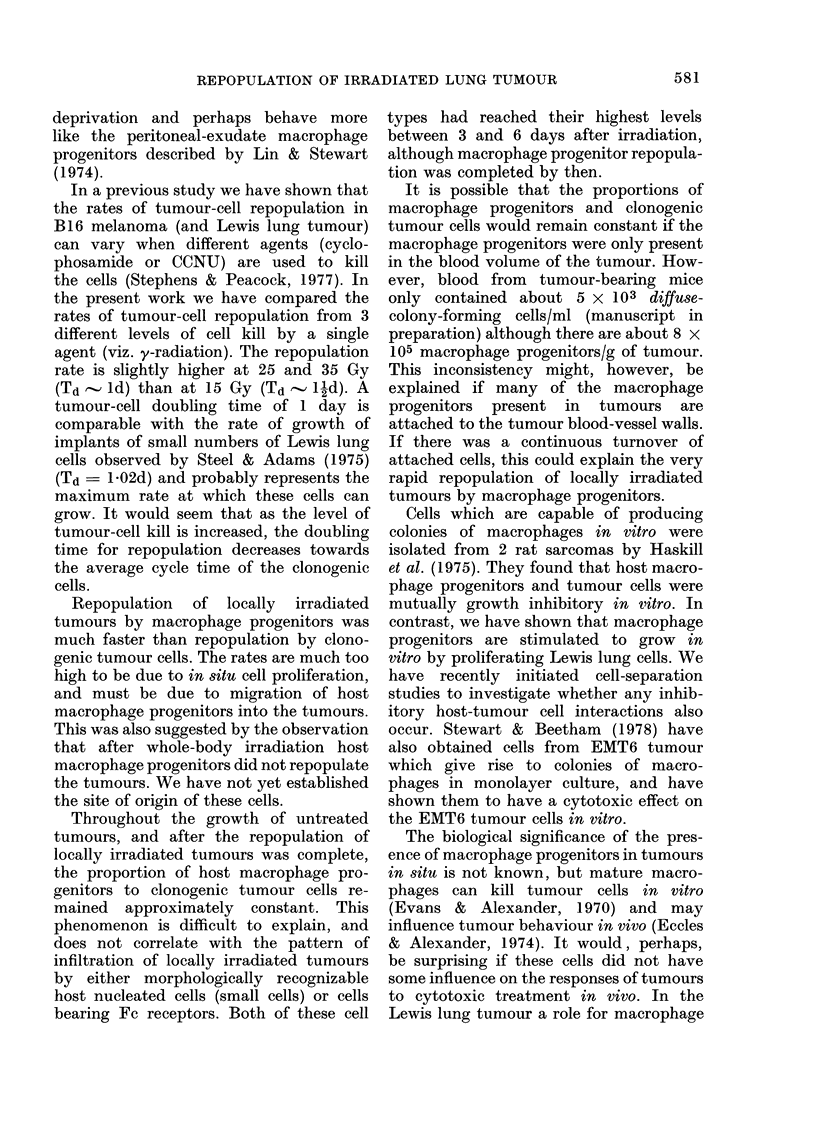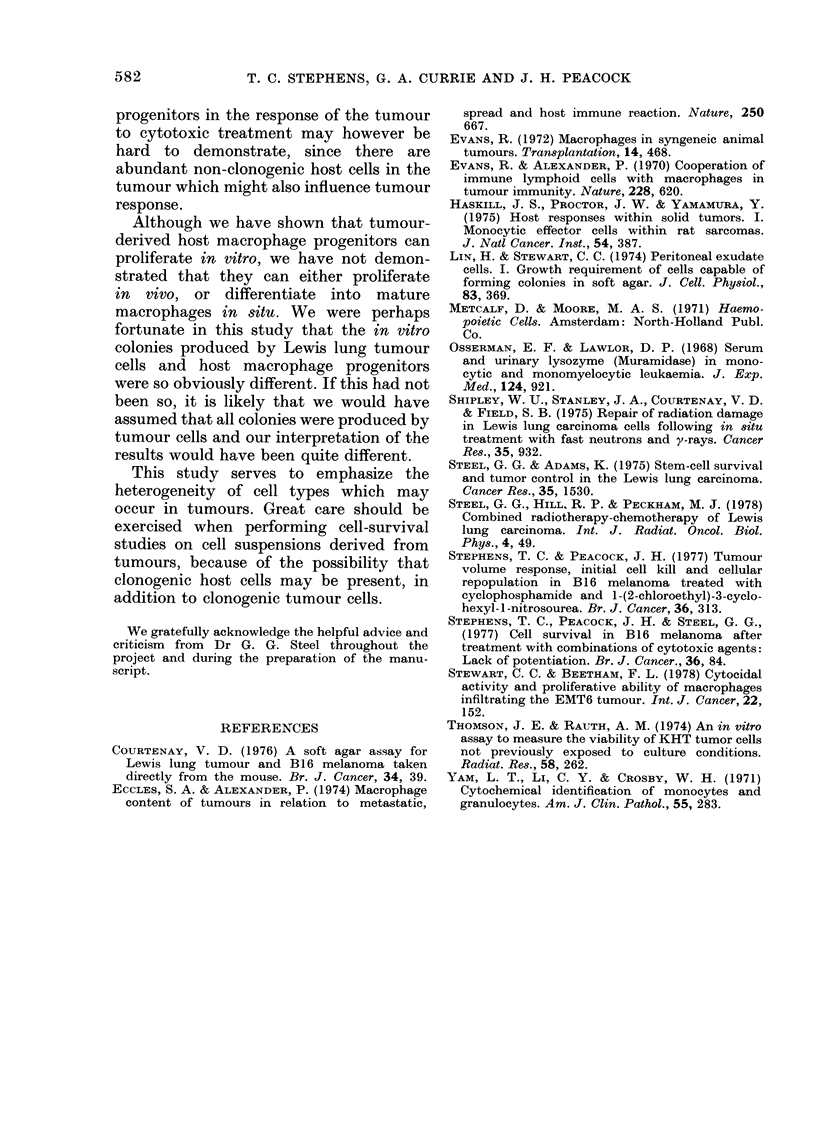# Repopulation of gamma-irradiated Lewis lung carcinoma by malignant cells and host macrophage progenitors.

**DOI:** 10.1038/bjc.1978.252

**Published:** 1978-11

**Authors:** T. C. Stephens, G. A. Currie, J. H. Peacock

## Abstract

**Images:**


*Br. J. Cancer* (1978) **38**, 573

### REPOPULATION OF $\gamma$-IRRADIATED LEWIS LUNG CARCINOMA BY MALIGNANT CELLS AND HOST MACROPHAGE PROGENITORS

T. C. STEPHENS, G. A. CURRIE* AND J. H. PEACOCK

*From the Departments of Radiotherapy Research and Tumour Immunology*, Institute of Cancer Research, Sutton, Surrey*

Received 13 June 1978 Accepted 18 August 1978

**Summary.—Cellular repopulation in Lewis carcinoma irradiated with $^{60}$Co $\gamma$-rays was examined by performing sequential cell-survival estimations using an *in vitro* soft-agar-colony assay. Following local irradiation (15–35 Gy) two distinct types of colony were seen: *compact* colonies with tightly packed cells and *diffuse* colonies with widely dispersed cells. Maximal *diffuse* colony formation *in vitro* was only obtained in the simultaneous presence of adequate numbers of *compact* colonies. After whole-body irradiation only *compact* colonies were observed.**

**Only cell-survival data from *compact* colony counts correlated with cell survival estimated by the lung colony assay and we conclude that *compact* colonies are produced by clonogenic tumour cells. Cytochemical and immunological evidence showed that *diffuse* colonies were composed of macrophages. After local irradiation the initial kill of clonogenic tumour cells was dose dependent. At each dose level, repopulation began immediately and proceeded with a doubling time of about 1 day. Macrophage colony-forming cells (macrophage progenitors) per tumour were initially reduced by about 3 decades, but recovered very rapidly to reach pretreatment levels within 2 days.**

**We conclude that at least two populations of clonogenic cells are present in Lewis lung carcinoma, tumour cells that repopulate irradiated tumours by *in situ* proliferation and host-macrophage progenitors that repopulate locally irradiated tumours by infiltration. The hazards of confusing host and tumour cell colonies in *in vitro* assay systems are stressed.**

THE GROWTH of malignant cells as colonies in soft agar has been used to examine cell survival and repopulation in tumours after experimental treatments (*e.g.* Thomson & Rauth, 1974; Shipley *et al.*, 1975; Stephens & Peacock, 1977).

Although host-derived cells such as fibroblasts will not form colonies in soft agar, haemopoietic stem cells will. Since many tumours contain a variety of host cells including those of haemopoietic origin (Evans, 1972; Haskill *et al.*, 1975) the possible contribution of clonogenic host cells in soft agar assays requires closer examination.

This paper describes studies on the Lewis lung tumour using a soft-agar assay. The experiments were designed to study the repopulation of locally irradiated tumours by clonogenic tumour cells, but also to demonstrate the presence of normal host colony-forming cells within the tumours. Changes in the numbers of these clonogenic host cells after irradiation, have been studied, and we have attempted to identify the clonogenic host cells. Their possible influence on the assessment of tumour cell survival by soft-agar-colony assays is discussed.

### MATERIALS AND METHODS

*Mice and tumour.*—Lewis lung carcinoma was maintained by i.m. transplantation in C57BL mice of the Institute of Cancer

Research colony. The procedure used to prepare a tumour brei and its implantation into gastrocnemius muscles of recipient mice, has been described by Steel & Adams (1975). Tumours were only implanted into the left hind leg, and were used for experiments 7 to 8 days after implantation, when they reached a weight of ~ 0·15 g.

*Irradiation.*—A $^{60}$Co gamma source was used. Radiation was either administered to the whole body by constraining conscious mice in perforated perspex boxes, or locally to the tumour by anaesthetizing the mice with 90 mg/kg of Saffan (Glaxo, Brentford, Middlesex) and using the irradiation jig described by Steel *et al.* (1978). A dose rate of ~ 3 Gy/min was used for both whole-body and local irradiations. Unconscious mice were kept in a warm-air environment, which prevented their body temperature from falling below 36°C during irradiation and until they regained consciousness.

*Cell suspensions.*—Cell suspensions were prepared from pooled samples of 2 i.m. Lewis lung tumours (taken from different animals). A method similar to that described for B16 melanoma by Stephens *et al.* (1977) was used to disaggregate the tissue. The only difference was that the duration of the second trypsinization was reduced from 45 to 20 min. Cell suspensions were counted by haemocytometer. Two populations of nucleated cells could be distinguished on the basis of size, large cells (mean diam 14·8 $\mu$m, s.d. 2·4) and small cells (mean diam 9·1 $\mu$m, s.d. 1·6). The overlap between the populations was not more than 10%. Each population was counted separately and the cell yield per g of untreated tumour was $8{\cdot}5 \times 10^7$ (s.d. $2{\cdot}8 \times 10^7$) for the large cells and $1{\cdot}35 \times 10^7$ (s.d. $0{\cdot}49 \times 10^7$) for the small cells. Vital staining with Trypan Blue indicated that the viability of each population was always > 95%.

*Cell survival.*—The survival of cells derived from Lewis lung tumours was assayed *in vitro* using the soft-agar-colony assay developed by Courtenay (1976). The dilution of cell suspensions for plating was based upon the counts of large cells. Cultures were incubated at 37°C in an atmosphere of 5% $O_2$, 5% $CO_2$ and 90% $N_2$ for ~ $1\frac{1}{2}$ weeks, and then colonies of more than 50 cells were counted. Plating efficiencies were calculated as PE = number of colonies scored/number of cells plated. Studies using the lung colony assay followed the method of Shipley *et al.* (1975).

For each treatment group the mean tumour weight, cell yield/tumour (tumour weight × cell yield/g) and yield of colony-forming cells/tumour (cell yield/tumour × PE) was calculated. The surviving fraction was calculated as SF = PE treated/PE control.

*Cell identification techniques.*—(i) Samples of cell suspensions, or cells from agar colonies, were gently centrifuged with IgG antibody-coated sheep erythrocytes (EA) at room temperature, and after resuspension the rosettes were counted under phase contrast, as a percentage of the total nucleated cells. Any cell bearing 3 or more adherent red cells was counted as a rosette. The same cell suspensions were also incubated at 37°C for 30 min and re-examined for phagocytosis.

(ii) Cells from agar colonies were allowed to adhere to glass microscope slides by incubation at 37°C for 1 h in serum-free medium. After examination for adherence, the cells were treated with 0·1% trypsin for 30 min. The cells not removed by trypsinization were then re-tested for the presence of Fc receptors.

(iii) Lysosyme in culture media was assayed by the lysoplate technique (Osserman & Lawlor, 1968).

(iv) Cell preparations were stained for non-specific esterase and for chloroacetate esterase using the methods of Yam *et al.* (1971).

### RESULTS

### *Colony morphology and growth requirements*

Two types of colony with distinct morphology were produced when the cells obtained from Lewis lung tumours were grown in soft agar. The majority of colonies from untreated tumours consisted of tightly packed cells, and a typical *compact* colony is shown in Fig. 1A. However, there were also a few colonies in which the cells were much more diffusely spread through the agar and a typical *diffuse* colony is shown in Fig. 1B.

After local irradiation the relative proportions of *compact* and *diffuse* colonies changed markedly, and the number of *diffuse* colonies was dependent on the number of *compact* colonies. Ten or more *compact* colonies per dish led to maximal *diffuse*-colony growth, but with less than 10, a cluster of *diffuse* colonies formed round each *compact* colony. When there

FIG. 1.—Colony types obtained when cells derived from untreated or locally irradiated Lewis lung carcinomas are grown in soft agar. A, *compact*; B, *diffuse*.

were no *compact* colonies, there were no *diffuse* colonies. Maximal *diffuse*-colony growth was restored in such cases by the addition of 100 cells from an untreated tumour, which would form 15–30 *compact* colonies but only 1–5 *diffuse* colonies.

*Identification of cells within colonies*

Lewis lung-tumour cells produce artificial metastases in mouse lungs when they are injected i.v., and a lung colony assay was developed by Shipley *et al.* (1975). An experiment was therefore performed to compare cell survival estimated by the lung colony assay and the *in vitro* assay, as this might indicate which type of *in vitro* colony was derived from tumour cells. Dose-response curves for $\gamma$-radiation, were determined from lung-colony counts, counts of *compact* colonies obtained *in vitro* after whole-body or local irradiation, and counts of *diffuse* colonies after local irradiation. After whole-body irradiation no diffuse colonies were seen. Fig. 2 shows the relationship between radiation dose and the fractions per tumour, of the different types of colony-forming cells which survived treatment. Assays were performed 24 h after irradiation.

The lung-colony data correlated closely with the *compact*-colony data, but not with the *diffuse*-colony data. This correlation strongly supports the conclusion that Lewis lung-tumour cells gave rise to *compact* colonies in the soft-agar assay. A best-fit line was drawn by eye through the lung-colony and *compact*-colony data, and the $D_0$ was 4·2 Gy.

*Diffuse* colonies were removed from the agar by a Pasteur pipette which had been drawn out into a capillary with a diameter less than that of a colony. In this way, only a very small amount of agar was obtained with the cells, and when they were placed in a drop of Hams F12 culture medium on a microscope slide they quickly adhered to the glass surface ($\sim$ 15 min) and could not be removed

FIG. 2.—The dose-survival curves of cells derived from Lewis lung tumours irradiated *in situ* with $^{60}$CO γ-rays. Tumours were either irradiated locally and cell survival was calculated from *in vivo* lung colony counts (■), *in vitro compact* colony counts (○) and *in vitro diffuse* colony counts (□), or irradiation was administered to the whole body and cell survival was calculated from *in vitro compact* colony counts (△). Assays were performed 24 h after irradiation in each case.

FIG. 3.—Growth curves of untreated Lewis lung tumours over the size range 0·1 to 3 g. Tumour weight (▲), total number of tumour cells per tumour, obtained by trypsinization (●) and number of clonogenic tumour cells (forming *compact* colonies) per tumour (○). Pooled data from 3 experiments.

by trypsinization. They all formed EA rosettes at room temperature, indicating the presence of Fc receptors, and were able to phagocytose opsonized sheep RBCat 37°C. The cells were mononuclear, stained strongly for non-specific esterase, but did not stain for chloroacetate esterase. There was also a correlation between the number of *diffuse* colonies in a culture dish and the concentration of lysozyme in the agar, but no correlation between the number of *compact* colonies and lysozyme level. Thus, we conclude that the cells in *diffuse* colonies are mononuclear phagocytes (macrophages) and we will refer to the cells which form colonies of macrophages as macrophage progenitors.

Cells from compact colonies did not bind EA or phagocytose opsonized RBC. Although they stain strongly for non-specific esterase, it is clear that they are not cells of the monocyte-macrophage series.

*Tumour-cell repopulation after local irradiation*

Tumour-cell repopulation after local irradiation of Lewis lung tumours with 15, 25 and 35 Gy of γ-rays, was followed by performing sequential soft-agar assays of cell survival and counting only *compact* colonies. Fig. 3 shows the growth curve of untreated tumours (solid triangles) from the size at which they were normally irradiated (0·15 g) until they reached about 3 g. The initial volume-doubling time was 2 days, followed by a gradual retardation. Total tumour-cell counts per tumour (*solid circles* in Fig. 3) were derived from the numbers of large cells in cell suspensions. These cells do not possess Fc receptors, comprise 85–90% of the total nucleated cell population obtained from untreated tumours, and

FIG. 4.—Cellular repopulation in Lewis lung tumour irradiated locally with 15 Gy of $\gamma$-rays. Symbols as in Fig. 3. Pooled data from 3 experiments.

FIG. 5—Cellular repopulation in Lewis lung tumour irradiated locally with 25 Gy of $\gamma$-rays. Symbols as in Fig. 3. Pooled data from 3 experiments.

their numbers follow closely the tumour-weight change. Only 10–25% of these cells are capable of producing *compact*-colonies in the soft-agar assay. The numbers of *compact* colony-forming cells (clonogenic tumour cells) per tumour are also shown in Fig. 3 and closely follow the change in tumour weight.

After 15 Gy, Lewis lung tumours continued to increase in weight for about 2 days, growth then stopped for about 2 days, but was later resumed (Fig. 4). There was no shrinkage below the initial tumour weight. The total number of tumour cells per tumour behaved in a similar way to the tumour weight. The number of colony-forming tumour cells per tumour was initially depressed by about $1\frac{1}{2}$ decades, repopulation began within 2 days, and proceeded with an average doubling time of about $1\frac{1}{2}$ days. Repopulation was complete by about 8 days.

At the higher dose of 25 Gy (Fig. 5) the response of tumour weight was very similar to that seen at 15 Gy, except that the period of growth delay was greater (about 5 days). The total number of tumour cells per tumour again closely followed tumour weight. The number of colony-forming tumour cells per tumour was initially depressed by about 3 decades, repopulation occurred with an average doubling time of about 1 day and was complete by about Day 11.

After exposure to 35 Gy (Fig. 6) tumours again continued to grow for about 2 days, but then began to shrink reaching a minimum size ($\sim$50% of pretreated weight) around Day 10. The total growth delay was about 13 days, and the tumours regrew with a doubling time of about 1 day. The total number of tumour cells per tumour showed a similar response to tumour-weight change. Colony-forming tumour cells were not detected during the 5 days immediately after irradiation, but repopulation by these cells occurred between Days 5 and 16, with an average doubling time of about 1 day.

FIG. 6.—Cellular repopulation in Lewis lung tumour irradiated locally with 35 Gy of γ-rays. Symbols as in Fig. 3. Pooled data from 3 experiments.

FIG. 7.—Macrophage progenitors per tumour during the growth of untreated Lewis lung tumours (A), and repopulation after local irradiation with 15 Gy (B), 25 Gy (C) and 35 Gy (D). Data from diffuse-colony counts made during the tumour-cell repopulation studies shown in Fig 3, 4, 5 and 6.

### *Macrophage-progenitor repopulation after irradiation*

The cell suspensions obtained during the tumour-cell repopulation studies were also assayed for macrophage progenitors. In the first experiment several dilutions of each cell suspension were prepared, and in subsequent experiments dilutions which would give 50–200 *diffuse* colonies per dish were used.

In these studies care was taken to ensure maximal *diffuse*-colony growth. Where only a few *compact* colonies were expected on a dish, 100 extra untreated cells were added to increase the number of *compact* colonies.

The numbers of macrophage progenitors obtained from untreated tumours are shown in Fig. 7A. The increase in macrophage-progenitor number closely followed the tumour growth curve shown in Fig. 3. The yield of macrophage progenitors from untreated tumours was about $8 \times 10^5$/g. compared to about $10^7$ colony-forming *tumour cells*/g (a ratio of 1:12·5).

The responses of macrophage progenitors to local irradiation with 15, 25 and 35 Gy are shown in Fig. 7B, C and D respectively. In each case, the number of macrophage progenitors per tumour was initially depressed by 2–3 decades, but recovered very rapidly to about $2 \times 10^5$, the control level on Day 2. Thereafter, at each dose the change in the number of macrophage progenitors appeared to follow the change in tumour weight (i.e. the yield of macrophage progenitors was roughly constant at about $8 \times 10^5$/g).

During the first few hours after local irradiation, the change in the surviving fraction of macrophage progenitors was very rapid, as shown in Fig. 8. The proportion of clonogenic macrophage progenitors increased from about $10^{-3}$ to $10^{-1}$ in less than 4h.

FIG. 8.—Changes in surviving fraction of macrophage progenitors in Lewis lung tumours during the 5h period immediately after local irradiation with 15, 25 and 35 Gy of $\gamma$-rays.

*Cellular content of suspensions derived from tumours*

About 10–15% of the nucleated cells in suspensions derived from untreated tumour were small, and we believe them to be of host origin. Between 35 and 50% of these small cells had Fc receptors. If macrophage progenitors are small cells, then their PE (number of colony-forming cells per tumour/number of small cells per tumour) would be about 6%. If they also have Fc receptors, their PE might be as high as 20%.

After local irradiation of tumours, the proportions of tumour-derived host cells (small) and cells with Fc receptors both increased markedly (Fig. 9A, B) with time after treatment.

At each dose the percentage of host cells in the cell suspensions rose sharply to a dose-dependent maximum on Day 3 (Fig. 9A). By Day 11, the proportion of host cells in suspensions derived from tumours receiving 15 Gy has returned to control level, at 25 Gy it was approaching the untreated level, but at 35 Gy much less recovery had occurred.

From Fig. 9B it is clear that the majority of the cells which migrate into tumours after local irradiation have Fc receptors. A very similar pattern of response was also obtained when lysozyme assays were performed on cell suspensions (data not shown).

### DISCUSSION

Cell suspensions from untreated or locally irradiated Lewis lung tumours contain two populations of cells, which are capable of forming colonies *in vitro* which can be easily distinguished by their morphology. The growth of *diffuse* colonies depended on the simultaneous presence within a culture dish of compact colonies, and at least 10 *compact* colonies per dish were required to achieve maximal *diffuse*-colony growth. Variations in the densities of either "large" or "small" cells per dish were inevitable in this study, since the proportions of each cell type in suspensions varied independently. We decided to maintain a level of $10^4$ large cells per dish by adding lethally irradiated large cells to dishes where less than $10^4$ large cells from the test suspension were plated. We have no evidence to suggest that the small-cell density has any influence on the clonal growth of either tumour cells or macrophage progenitors.

The comparison of cell survival measured using the *in vivo* lung colony assay and the *in vitro* soft-agar assay strongly suggests that tumour cells give rise to *compact* colonies in agar. Furthermore, *diffuse* colonies have been shown by cytochemical techniques to have the characteristics of host macrophages, although it has not been established

FIG. 9.—Changes in the proportions of host nucleated cells (A) and cells with Fc receptors (B) in cell suspensions from locally irradiated Lewis lung tumours. In each case the symbols are (●) untreated, (○) 15 Gy, (△) 25 Gy and (□) 35 Gy.

whether the cells that give rise to colonies of macrophages are themselves macrophages or some more primitive progenitor.

It is well established that the marrow-derived cell known as CFU-C is a progenitor of macrophages (and granulocytes) *in vitro* in soft agar, but proliferation only occurs in the presence of a diffusible colony-stimulating factor (CSF) (Metcalf & Moore, 1971). This factor has been obtained from a variety of cell types, including lymphomas and leukaemias. A plausible explanation of the observed dependency of *diffuse*-colony growth on the simultaneous presence of *compact* colonies might be that the macrophage progenitors present in tumours required a colony-stimulating factor, which is produced by proliferating Lewis lung cells. Recently we have confirmed that host macrophage progenitors derived from tumours, and CFU-C's from mouse marrow, are both stimulated to form colonies of macrophages by culture medium *conditioned* over monolayers of Lewis lung cells and by pregnant-mouse uterus extract (PMUE) a potent source of CSF (manuscript in preparation). It is possible that optimal macrophage-progenitor growth has not been achieved in the experiments described in this paper because the concentration of CSF might only have risen slowly to effective levels as the tumour colonies grew *in vitro*. Metcalf has shown that most CFU-C's fail to produce colonies if they are incubated for 2–3 days in the absence of CSF (Metcalf & Moore, 1971, p. 392). We have compared the numbers of macrophage colonies produced when CSF was supplied either by growing tumour colonies, or by the addition of PMUE at the time of plating. Assays were performed at various times after treatment with 15, 25 and 35 Gy. The patterns of response were similar with each source of CSF, but the macrophage-colony number was about twice as great when PMUE was used. Since tumour-colony growth is not apparent in cultures for several days, it seems likely that our macrophage progenitors might be much less sensitive to CSF

deprivation and perhaps behave more like the peritoneal-exudate macrophage progenitors described by Lin & Stewart (1974).

In a previous study we have shown that the rates of tumour-cell repopulation in B16 melanoma (and Lewis lung tumour) can vary when different agents (cyclophosamide or CCNU) are used to kill the cells (Stephens & Peacock, 1977). In the present work we have compared the rates of tumour-cell repopulation from 3 different levels of cell kill by a single agent (viz. $\gamma$-radiation). The repopulation rate is slightly higher at 25 and 35 Gy ($T_d \sim 1d$) than at 15 Gy ($T_d \sim 1\frac{1}{2}d$). A tumour-cell doubling time of 1 day is comparable with the rate of growth of implants of small numbers of Lewis lung cells observed by Steel & Adams (1975) ($T_d = 1{\cdot}02d$) and probably represents the maximum rate at which these cells can grow. It would seem that as the level of tumour-cell kill is increased, the doubling time for repopulation decreases towards the average cycle time of the clonogenic cells.

Repopulation of locally irradiated tumours by macrophage progenitors was much faster than repopulation by clonogenic tumour cells. The rates are much too high to be due to *in situ* cell proliferation, and must be due to migration of host macrophage progenitors into the tumours. This was also suggested by the observation that after whole-body irradiation host macrophage progenitors did not repopulate the tumours. We have not yet established the site of origin of these cells.

Throughout the growth of untreated tumours, and after the repopulation of locally irradiated tumours was complete, the proportion of host macrophage progenitors to clonogenic tumour cells remained approximately constant. This phenomenon is difficult to explain, and does not correlate with the pattern of infiltration of locally irradiated tumours by either morphologically recognizable host nucleated cells (small cells) or cells bearing Fc receptors. Both of these cell types had reached their highest levels between 3 and 6 days after irradiation, although macrophage progenitor repopulation was completed by then.

It is possible that the proportions of macrophage progenitors and clonogenic tumour cells would remain constant if the macrophage progenitors were only present in the blood volume of the tumour. However, blood from tumour-bearing mice only contained about $5 \times 10^3$ *diffuse*-colony-forming cells/ml (manuscript in preparation) although there are about $8 \times 10^5$ macrophage progenitors/g of tumour. This inconsistency might, however, be explained if many of the macrophage progenitors present in tumours are attached to the tumour blood-vessel walls. If there was a continuous turnover of attached cells, this could explain the very rapid repopulation of locally irradiated tumours by macrophage progenitors.

Cells which are capable of producing colonies of macrophages *in vitro* were isolated from 2 rat sarcomas by Haskill *et al.* (1975). They found that host macrophage progenitors and tumour cells were mutually growth inhibitory *in vitro*. In contrast, we have shown that macrophage progenitors are stimulated to grow *in vitro* by proliferating Lewis lung cells. We have recently initiated cell-separation studies to investigate whether any inhibitory host-tumour cell interactions also occur. Stewart & Beetham (1978) have also obtained cells from EMT6 tumour which give rise to colonies of macrophages in monolayer culture, and have shown them to have a cytotoxic effect on the EMT6 tumour cells *in vitro*.

The biological significance of the presence of macrophage progenitors in tumours *in situ* is not known, but mature macrophages can kill tumour cells *in vitro* (Evans & Alexander, 1970) and may influence tumour behaviour *in vivo* (Eccles & Alexander, 1974). It would, perhaps, be surprising if these cells did not have some influence on the responses of tumours to cytotoxic treatment *in vivo*. In the Lewis lung tumour a role for macrophage progenitors in the response of the tumour to cytotoxic treatment may however be hard to demonstrate, since there are abundant non-clonogenic host cells in the tumour which might also influence tumour response.

Although we have shown that tumour-derived host macrophage progenitors can proliferate *in vitro*, we have not demonstrated that they can either proliferate *in vivo*, or differentiate into mature macrophages *in situ*. We were perhaps fortunate in this study that the *in vitro* colonies produced by Lewis lung tumour cells and host macrophage progenitors were so obviously different. If this had not been so, it is likely that we would have assumed that all colonies were produced by tumour cells and our interpretation of the results would have been quite different.

This study serves to emphasize the heterogeneity of cell types which may occur in tumours. Great care should be exercised when performing cell-survival studies on cell suspensions derived from tumours, because of the possibility that clonogenic host cells may be present, in addition to clonogenic tumour cells.

We gratefully acknowledge the helpful advice and criticism from Dr G. G. Steel throughout the project and during the preparation of the manuscript.

### REFERENCES

Courtenay, V. D. (1976) A soft agar assay for Lewis lung tumour and B16 melanoma taken directly from the mouse. *Br. J. Cancer*, **34**, 39.

Eccles, S. A. & Alexander, P. (1974) Macrophage content of tumours in relation to metastatic, spread and host immune reaction. *Nature*, **250** 667.

Evans, R. (1972) Macrophages in syngeneic animal tumours. *Transplantation*, **14**, 468.

Evans, R. & Alexander, P. (1970) Cooperation of immune lymphoid cells with macrophages in tumour immunity. *Nature*, **228**, 620.

Haskill, J. S., Proctor, J. W. & Yamamura, Y. (1975) Host responses within solid tumors. I. Monocytic effector cells within rat sarcomas. *J. Natl Cancer. Inst.*, **54**, 387.

Lin, H. & Stewart, C. C. (1974) Peritoneal exudate cells. I. Growth requirement of cells capable of forming colonies in soft agar. *J. Cell. Physiol.*, **83**, 369.

Metcalf, D. & Moore, M. A. S. (1971) *Haemopoietic Cells*. Amsterdam: North-Holland Publ. Co.

Osserman, E. F. & Lawlor, D. P. (1968) Serum and urinary lysozyme (Muramidase) in monocytic and monomyelocytic leukaemia. *J. Exp. Med.*, **124**, 921.

Shipley, W. U., Stanley, J. A., Courtenay, V. D. & Field, S. B. (1975) Repair of radiation damage in Lewis lung carcinoma cells following *in situ* treatment with fast neutrons and $\gamma$-rays. *Cancer Res.*, **35**, 932.

Steel, G. G. & Adams, K. (1975) Stem-cell survival and tumor control in the Lewis lung carcinoma. *Cancer Res.*, **35**, 1530.

Steel, G. G., Hill, R. P. & Peckham, M. J. (1978) Combined radiotherapy-chemotherapy of Lewis lung carcinoma. *Int. J. Radiat. Oncol. Biol. Phys.*, **4**, 49.

Stephens, T. C. & Peacock, J. H. (1977) Tumour volume response, initial cell kill and cellular repopulation in B16 melanoma treated with cyclophosphamide and 1-(2-chloroethyl)-3-cyclohexyl-1-nitrosourea. *Br. J. Cancer*, **36**, 313.

Stephens, T. C., Peacock, J. H. & Steel, G. G., (1977) Cell survival in B16 melanoma after treatment with combinations of cytotoxic agents: Lack of potentiation. *Br. J. Cancer.*, **36**, 84.

Stewart, C. C. & Beetham, F. L. (1978) Cytocidal activity and proliferative ability of macrophages infiltrating the EMT6 tumour. *Int. J. Cancer*, **22**, 152.

Thomson, J. E. & Rauth, A. M. (1974) An *in vitro* assay to measure the viability of KHT tumor cells not previously exposed to culture conditions. *Radiat. Res.*, **58**, 262.

Yam, L. T., Li, C. Y. & Crosby, W. H. (1971) Cytochemical identification of monocytes and granulocytes. *Am. J. Clin. Pathol.*, **55**, 283.